# Understanding the impact of COVID-19 on the informal sector workers in Bangladesh

**DOI:** 10.1371/journal.pone.0266014

**Published:** 2022-03-31

**Authors:** Nahrin Rahman Swarna, Iffat Anjum, Nimmi Nusrat Hamid, Golam Ahmed Rabbi, Tariqul Islam, Ezzat Tanzila Evana, Nazia Islam, Md. Israt Rayhan, KAM Morshed, Abu Said Md. Juel Miah

**Affiliations:** 1 Advocacy for Social Change, BRAC Centre, Mohakhali, Dhaka, Bangladesh; 2 Institute of Statistical Research and Training (ISRT), University of Dhaka, Dhaka, Bangladesh; Universiti Malaya, MALAYSIA

## Abstract

The COVID-19 pandemic put dents on every sector of the affected countries, and the informal sector was no exception. This study is based on the quantitative analyses of the primary data of 1,867 informal workers of Bangladesh to shed light on the impact of the pandemic-induced economic crisis on this working class. The survey was conducted between 8 July and 13 August 2020 across the eight administrative divisions of the country. Analysis points out that about ninety percent of these workers faced an income and food expenditure drop during the lockdown. The effect was higher in males, particularly among the urban-centric and educated males engaged in services and sales. The findings suggest that policy support is needed for the informal workers to face such a crisis.

## 1. Introduction

The COVID-19 pandemic caused over 5.9 million deaths globally [1] and resulted in an economic crisis for almost all countries. The safety measures taken to restrain the spread of the virus (such as quarantine, travel restrictions, closure of educational and business institutions, reduced public gatherings) disrupted economic activities worldwide. The pandemic was predicted to harm the low-income population, especially the informal sector workers with vulnerable employment and minimal health or social safety [2, 3]. About 1.6 billion workers in the informal economy faced turmoil due to COVID-19 [4]. About 60% experienced a drastic drop down in their earning. Forty two percent women and 32% men informal workers, besides those of the micro-industries (employing less than 10 persons), were the hardest hit [4]. The slump in demand for goods and services resulting from the pandemic and its concomitant restrictive measures caused a loss of income and jobs for informal workers, whose livelihood depends on consumption-driven economic activities. There were some major factors behind the reduced demand for consumption during this crisis. First, restriction on movement caused demand for non-essential goods to decline. Second, the lockdown and the pandemic hurt peoples’ earning capacity, which reduced their consumption expenditure [5]. In the time of high demand, informal sector workers got more work opportunities; on the other hand, when demand was low, firms used to reduce costs by laying off informal employees and terminating purchase orders given to informal enterprises [6].

There are two opposing views regarding the impact of an economic crisis on the informal sector–the optimistic view suggests that the informal sector may work as a safety net for the economy during a crisis; while the pessimistic view suggests that the informal sector can be badly impacted during a crisis due to income uncertainty and lack of social security support from the government [7, 8]. The devastating impact of the pandemic on household income has been highlighted in several studies across countries. More than two-thirds of the respondents from the informal sector in Kenya and Uganda depicted income downfall due to the COVID-19 crisis [9]. Households in rural Uganda were found to have experienced a 60% fall in household non-farm income as enterprises lost profits and workers suffered wage loss [10]. In the Democratic Republic of Congo, 84% of respondents reported a decline in income; per capita food expenditure dropped by almost 40% for COVID-19, caused mainly by jobs and wage losses [11]. The pandemic was found to have a devastating impact on informal sector workers in Thailand. Around 95% of the responding informal sector workers in a survey of 384 samples reported that they experienced a drastic fall in income during the pandemic [12]. Another study conducted in six developed countries suggested that women experienced a 24% higher risk of losing their job permanently compared to men because of the Coronavirus outbreak [13].

The lack of social protection coverage for informal sectors made it more difficult for developing countries with large informal sectors to build resilience against the Covid-19 pandemic and recover quickly from the economic fallout [14]. Leyva and Urrutia [15] studied the labor markets dynamics in five Latin American countries and found that the nature of informal employment poses challenges for pandemic management. The study also found a drop in informal employment during the onset of the pandemic in these countries. Informal sector businesses in Uganda were adversely affected by the restriction on movement and lockdown imposed due to the pandemic [16]. The survey revealed that 36% of respondents from Uganda, 20% from Myanmar, and 15% from Nepal, lost regular income sources during the lockdown. In Kenya, urban dwellers had severe income downfall during the pandemic [17]. In Myanmar, 60% of households experienced work stoppage, of which 49% of owners of small enterprises shut down their operations [18, 19]. Informal sex workers faced unemployment risk due to social distancing, and their family members were deprived of care [20]. Furthermore, their regular health care service denial and violence rate were exacerbated [21]. The pandemic caused decline in aggregate consumption and a surge in informal unemployment in Colombia and Peru [22]. A strict lockdown dismantled street vending in India [23]. Even though the aid programs were stimulated to rebuild the economy, the illiterate informal sector workers experienced a lack of access to the Government relief provided during the pandemic [17].

Bangladesh went into a lockdown of over two months after the first case of Coronavirus was detected in March 2020. As informal sectors of developing countries worldwide face the grim economic crisis, Bangladesh was no different. With a large informal sector, the pandemic threatened to thwart the country’s economic development that took decades to achieve. According to BBS [24], 85.1% of the workforce, or 51.7 million people, are employed in the informal sector. This percentage was 86.2% in 2015–16 and 87.5% in 2010, reflecting a downward trend. More females (91.8%) are involved than males (82.1%) in this sector. 13.1 million urban workers (77.3%) are informal sector workers, whereas 38.6 million rural workers (88.1%) are informally employed. 95.4% of workers involved in agriculture are informal workers. The informal sector contributes 43% of the GDP [25, 26]. As per the Bangladesh Bureau of Statistics, the informal sector refers to unregistered private goods or service enterprises [27]. Such enterprises are unincorporated and mostly operate on a small scale. Employment under these sectors is casual, without any legal bindings, formal employment protection, or benefits. Production levels in the informal sector enterprises are low and the distinction between capital and labor is unclear [25]. Since the beginning of the pandemic, there have been attempts to collect microdata to understand the pandemic’s economic impact. The COVID-19 pandemic has disrupted the socio-economic situation of people and their livelihoods in Bangladesh [28–31]. A phone survey conducted in April 2020 found a 75% income drop for urban slum respondents and a 62% income drop for rural respondents [32]. Barkat [33] predicted that the economic shutdown due to pandemic might result in an unimaginable loss for an estimated 60 million low-income vulnerable people in Bangladesh and suggested a transfer payment amounting to BDT 81,000 crore or USD 9.49 billion to provide sustenance. A study [34] estimated an economywide loss of 11.1 million jobs during the lockdown of April-May 2020, while the job loss for the urban informal sector is estimated to be 1.08 million. Another survey [35] of 244 low-income people who were involved in informal work suggested that 50% of the respondents experienced diminished income, while 47% had their income reduced to zero during the lockdown. Both formal and informal businesses in Bangladesh were severely hampered during the lockdown [36]. A CPD-BILS [37] study identified workers involved in the informal sector as the most affected working group by the pandemic and pointed out that the recovery for the informal sector workers, self-employment and small and medium enterprises (SMEs) has been slow. However, the poorest cluster, which has little or no saving and relies on the informal sector, became the worst victim of economic turmoil and lockdown [38–41]. The ready-made garment (RMG) sector is playing an essential role in shifting the economic pattern in the informal job market in Bangladesh, particularly in empowering the less educated women [42]. Even the RMG sector has faced an economic recession during the COVID-19 pandemic due to a sudden decrease in the demand of the European market [43, 44]. A Government stimulus package was announced to revive the informal economy [45]. It might not reach the informal jobless workers and vulnerable women rather accessible to the industry owners [46]. An obvious deprivation of the intended beneficiaries and sometimes loopholes of the delivery channels often hamper the success of such interventions, as suggested by a study conducted in 140 developing countries [47]. Recent studies also focused that the crisis regulators were not effectively communicating information with whom are eligible for relief and how it can be availed [48, 49]. The crisis caused by the COVID-19 pandemic zoomed out the flip side of the coin, where the informal sector was the savior of Bangladesh’s economy during the 2008 global recession [50], requiring support to recover from the economic turmoil.

**Fig 1:**
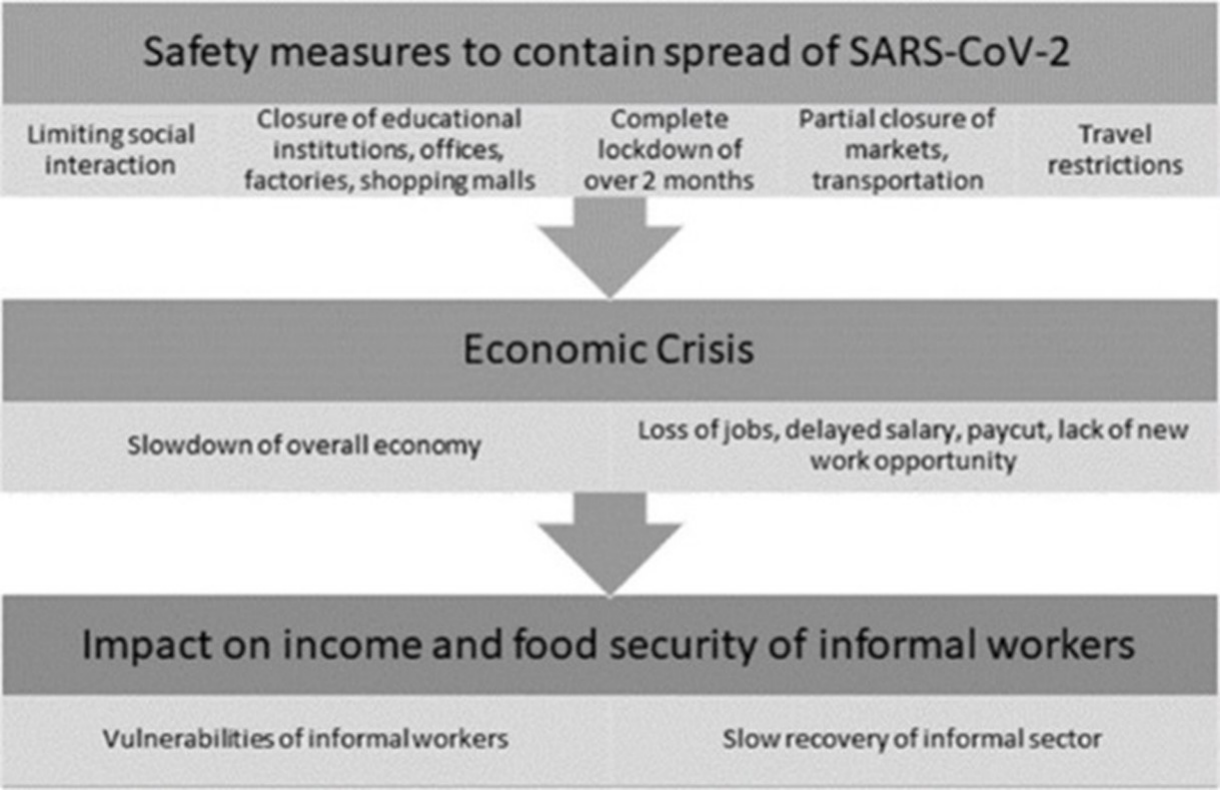
Conceptual framework.

While there have been a few studies focusing on the estimation of loss of jobs in the informal sector, a more coherent picture of the true sufferings of informal workers was needed. BRAC conducted this study to bridge the gap to bring quantitative evidence from the field on the impact of the pandemic on these workers. This study opted to identify the challenges informal workers faced and their coping strategies during the pandemic. As a major contributor to the economy, this study objective was to investigate the vulnerabilities of the informal sector workers due to the COVID-19. Another objective was to perform an elaborative analysis on income and food expenditure shortfall due to pandemic using some parameters, such as rural-urban differentials, gender identity, education, age, and types of work. Fig 1 depicted the conceptual framework of the study:

Research objectives and literature review are elaborated in the introduction section, the survey and sampling design of data collection and the methodology are explained in section two. Results and analyses are sequentially placed in section three and conclusion and policy suggestions are assigned in section four.

## 2. Materials and methods

### 2.1 Survey data

This study analyzed primary data using various methods and tools. The survey was conducted through telephone interviews between 8 July and 13 August 2020, included 1,867 informal sector workers across the eight administrative divisions of Bangladesh. The questionnaire was comprehensive in understanding the challenges, coping strategies and future plans. Considering that a very limited database of informal sector workers exists in the country, contact details (mobile phone numbers) were collected through district-level officials of BRAC. Of the respondents, 25% were BRAC beneficiaries.

In some cases, such as sex workers, sanitation and hotel restaurant workers, a snowball sampling technique was used to collect the contact details. The respondents were randomly selected from the compiled database. One of the major challenges faced during the survey was the availability of workers in the telephonic interview. Since contact details were collected from various sources, ensuring equal representation from each sub-sector was not possible. However, the Labor Force Survey [27] microdata reveals that 93.5% of informal workers have access to mobile phones, compared to 97.5% of formal workers. Anonymity was ensured for the respondents, and research objectives and purposes were well explained to them beforehand. This study used human subjects for a quantitative survey with their prior oral consent on the questionnaire; no minors were interviewed. This study also received ethical approval from the BRAC advocacy Internal Review Board (IRB).

The sample size was determined by the formula [51] of cross-sectional study: $n=\frac{pqz^{2}}{d^{2}}\times f\times k$, where, *n* is the sample size; *z* (1.96) is standard normal variate, for a 95% confidence interval; p (0.85) is an estimate of key indicator, 85% informal sector workers of working labor force [24]; *q* = 1 –*p*; *f* (1.3) is design effect, *k* (8) is the eight administrative divisions. The quantitative survey questionnaire was kept short, keeping telephone interviews in mind, and focused on the aftermath of the lockdown. Respondents were asked about their monthly income before (February) and during the COVID-19 (June), weekly food expenditure before and after lockdown, age, gender, area of residence, education, profession, perception whether lockdown hampered their income, coping strategies, from whom they received any aid or assistance. Food expenditure was later converted from weekly to monthly to adjust the inflation rate. The survey was initially conducted with 2,035 (sample size) informal workers; among them, 1,867 respondents reported their monthly income (8% missing cases). This study analyzed the economic downfall due to the lockdown and thus counted the completed survey for 1,867 respondents. Income and food expenditure data have been adjusted for inflation. Food price inflation rates were 4.97% and 6.54%, and overall inflation rates were 5.46% and 6.02% for February and June, respectively (the base year 2005–06) [52]. Individual income and food expenditure were asked because many of them resided near their workplace apart from the family. Data were analyzed through Stata/MP 17 software.

**Fig 2:**
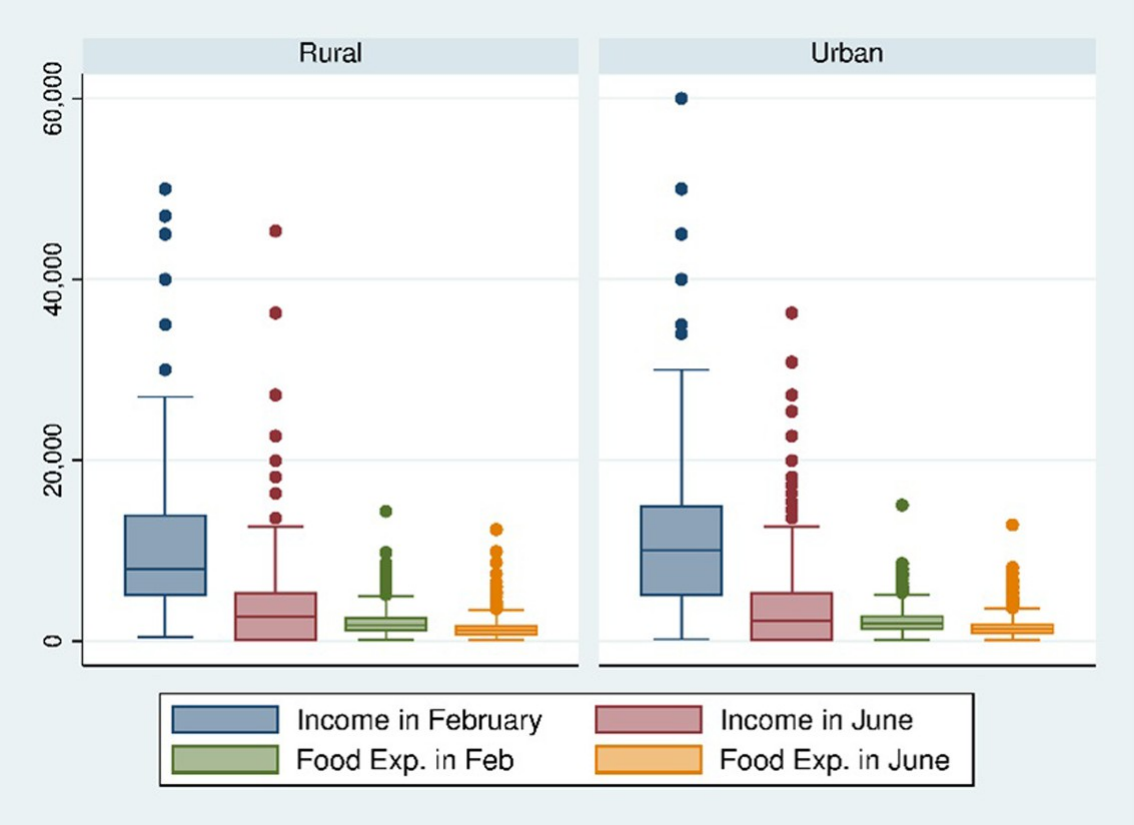
Box-plots for numerical income and food expenditure data.

Fig 2 showed box-plots of numeric variables: personal incomes in February and June, and food expenditures in two different months for rural-urban areas. Monthly income in February for rural areas depicts slightly positive skewed data; apart from that, overall data show the symmetric pattern and a higher quantity for urban areas. Also, median income dropped drastically from February to June for both areas. Food expenditures delineate symmetric patterns, though slightly high food expenses are shown before the pandemic and in urban areas.

### 2.2 Models

Analyses of this study initiated with the bivariate and graphical illustrations. Few parametric tests (independent t-test, paired t-test, ANOVA) were performed to check the association between the income and food expenditure gaps (February to June) and the socio-economic covariates. Ordinary least square estimates were found from the multiple linear regression with a forward-step inclusion of the covariates. With the post-estimation measures (Akaike and Bayesian information criteria) [53], the following model was depicted as the best fitted one: $y_{i}=\beta_{0}+\beta_{1}x_{i}+\beta_{2}x_{i}^{2}+\beta_{3}D_{1i}+\beta_{4}D_{2i}+\beta_{5}D_{3i}+\beta_{6}D_{4i}+\beta_{7}D_{5i}+e_{i}$, where, *y* is the income or food expenditure gap, *x* is the age of respondent, *D*_1_, *D*_2_, *D*_3_, *D*_4_, *D*_5_ are the gender, area, division, education and profession, respectively, *e* is a random error. Here the dependent variable *y* in the fixed-effect ordinary least square (OLS) is the per capita income or food-expenditure gap, measured by the amount in February (before lockdown) minus the amount in June (during lockdown). For a cross-sectional study, a heteroscedasticity check is necessary. Breusch-Pagan test [54] was performed to assess the homoscedasticity of error variances and robust standard errors are estimated for the regression parameters. Pearson chi-square test (expected cells are more than 5) was examined to check the independence of categorical variables. A Heckman OLS model was also analyzed to see whether there were any significant changes for the BRAC beneficiaries and other respondents; thus, the model was fitted as: $y_{i}=\beta_{0}+\beta_{1}x_{i}+\beta_{2}x_{i}^{2}+\beta_{3}D_{1i}+\beta_{4}D_{2i}+\beta_{5}D_{3i}+\beta_{6}D_{4i}+\beta_{7}D_{5i}+e_{i}$, where, *y*_*i*_ is observed if, $\delta_{0}+\delta_{1}x_{i}+\delta_{2}x_{i}^{2}+\delta_{3}D_{1i}+\delta_{4}D_{2i}+\delta_{5}D_{3i}+\delta_{6}D_{4i}+\delta_{7}D_{5i}+u_{i}$ > 0 and *u*_*i*_ and *e*_*i*_ have correlation *ρ*.

The probit model was also considered to find the associated covariates for the income and food expenditure downfalls. If there was a downfall of income or food expenditure gap, the model considered *y* = 1, and 0 for an increase in income or food expenditure from February to June. The model was fitted as: $\mathrm{mathvariant="normal">Pr}\left(y_{i}\neq 0|x_{i}\right)=\Phi \left(x_{i}\beta \right)$, where, Φ is the standard cumulative normal. A zero-inflated probit model was fitted because the income downfall rate was 98% and food expenditure dropdown rate was about 95%, here the downfall was taken as *y* = 0 [53].

## 3. Results

During COVID-19 lockdown, about 98% of informal workers experienced an income drop, and on average, the amount is about BDT 6,829 (US$ 80) from the sample of 1,867 workers. Informal workers were sampled randomly from the available list collected through BRAC local officials and local authorities. Those selected reflected, somehow, little dominance of urbanism (urban 60: rural 40). Gender ratio was almost equal for workers (male 52: female 48). Divisional ratio was addressed in terms of their working place; Dhaka occupied the highest proportion. Higher education (above secondary level) in the informal working class was low (8%); plant and machine operators had the lowest representation (5%) in the sampled data.

Table 1 displayed the income downfall was highest (in percentages) in the age group below 18 years. About 81% of the workers belonging to the age group 18–45 years, had an average income drop of BDT 6,937 (US$ 82). Male workers had a larger reduction of income in terms of absolute value compared to that of the females. The average decrease in income for the males was about BDT 7,506 (US$ 88), whereas for the females it was around BDT 6,093 (US$ 72). The reduction of males’ income in June compared to that in February was significantly higher than that of females’ at a 1 percent level of significance. Interestingly, while the percentage changes are considered, female workers (69%) faced more hardship compared to that of the male workers (61%). Urban informal sector workers had a significantly higher decrease in income compared to that of their rural counterparts, both in amount and in percentage [17, 56]. Sylhet division faced the highest (both in amount and percentages) income downfall. ANOVA test showed a significant difference in mean income for different professions and educational qualifications. In terms of income downfall, the percentage was higher for the workers without schooling (67%). Workers engaged with service and sales faced the highest income decrease both in amount (BDT 10112, US$ 119) and in percentages (68%).

**Table 1:** Distribution and association of socio-economic characteristics and income downfall.

Variables	Frequency (Percentage)	Average income drop in BDT*	Rate of income drop (percentage)	Test, p-value, decision (income drop from February to June)
**Overall**		6828.91	64.61	
**Age**				
<18	41 (2.01)	3304.03	67.47	ANOVA test, F-statistic = 4.92, p-value: <0.01, All means are not equal
18–45	1650 (81.08)	6936.78	64.64	
45+	344 (16.90)	6663.88	64.16	
**Gender**				
Male	972 (52.06)	7506.49	60.79	t-statistic = 4.66, p-value: <0.01, male>female
Female	895 (47.94)	6093.03	68.85	
**Area**				
Rural	739 (39.58)	6260.79	63.89	t-statistic = 3.03, p-value: <0.01, rural<urban
Urban	1128 (60.42)	7201.11	65.07	
**Division**				
Barishal	140 (7.50)	7145.93	65.23	ANOVA test, F-statistic = 5.16, p-value: <0.01, All means are not equal
Chattogram	428 (22.92)	6632.47	64.37	
Dhaka	518 (27.75)	7482.06	63.17	
Khulna	233 (12.48)	4837.95	57.91	
Mymensingh	124 (6.64)	7814.67	64.08	
Rajshahi	92 (4.93)	6346.43	68.69	
Rangpur	207 (11.09)	6492.75	70.09	
Sylhet	125 (6.70)	8084.89	71.56	
Education**				
No Schooling	518 (27.75)	5768.71	66.83	ANOVA test, F-statistic = 14.53, p-value: <0.01, All means are not equal
Primary	656 (35.14)	6424.91	63.96	
Secondary	550 (29.46)	7729.88	63.19	
Above Secondary	143 (7.66)	9057.41	64.95	
Profession***				
Service & sales	339 (18.16)	10111.74	67.58	ANOVA test, F-statistic = 38.45, p-value: <0.01, All means are not equal
Craft & trade	626 (33.53)	5839.67	63.84	
Plant & machine operator	89 (4.77)	7596.32	63.12	
Elementary occupation	813 (43.550	6137.75	64.11	

*USD 1 = BDT 85.

** When forming education categories for modeling purposes, workers who reported they cannot read have been categorized as “No schooling”; workers who can read and studied up to grade 5 as “Primary”; workers who studied beyond grade 5 but below higher secondary level as “Secondary”, and those with above secondary education as “Above secondary.”

***Profession category from BSCO [55]; Beauty parlor and salon workers, workers in shopping malls, grocery stores/tea stalls, and sex workers have been grouped under the “Service and sales workers” category; carpenter/mason, sanitation workers/plumbers, tailor, handicraft workers, and food processing workers have been grouped under “Craft and related trades workers”; rice mill workers and drivers of CNG/auto rickshaw have been grouped under “Plant and machine operators and assemblers”; agricultural workers, domestic help, construction workers, rickshaw/van pullers, hotel/restaurant workers, and hawkers have been grouped under “Elementary occupations”.

**Fig 3:**
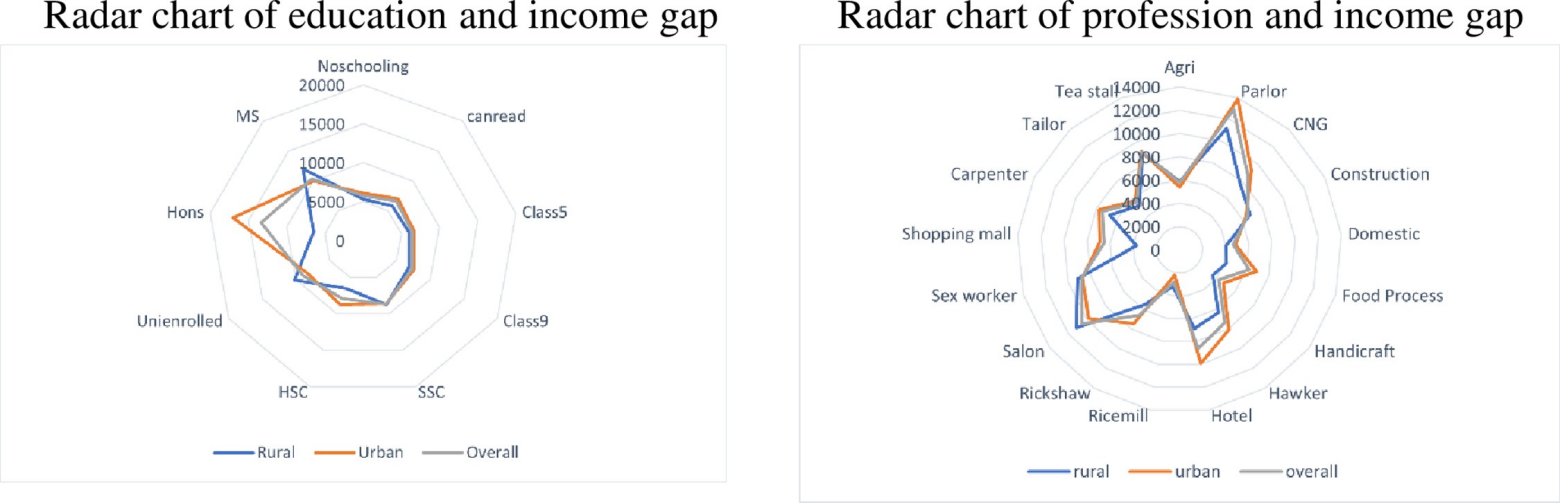
Radar charts of education and profession with the income gap.

Fig 3 illustrated the average downfall of income during the pandemic. The radar graph depicted a segregated picture of the variation of income downfall among the professional and education levels. Higher educated informal workers had a higher average loss (though the sample sizes for MS, Honors and University enrolled were only 12, 20 and 25 informal workers, respectively). Professional sectors that serve luxury or lesser-necessary-care service (beauty parlor, salon, hotel, sex workers) were the most affected [20, 21].

Table 2 demonstrated that the average food expenditure decrease was BDT 658 (US$ 8), about a 28% decrease, between February and June. The age group 18–45 years had the maximum food expenditure drop both in amount (BDT 673, US$ 8) and percentage (29%). Urban workers had a higher food expenditure shrinkage than that of their rural counterparts. Barishal division showed the maximum downfall of food expenditure (32%). Workers with no schooling had the lowest amount and percentage of food expenditure reduction. Informal workers in service and sales could hardly afford a 31% lesser food budget in the pandemic than that of the before-pandemic period.

**Table 2:** Distribution and association of socio-economic characteristics and food expenditure downfall.

Variables	Frequency (Percentage)	Average food exp. drop in BDT	Rate of food exp. drop in percentage	Test, p-value, decision (food expenditure drop February to June)
**Overall**		657.68	28.13	
**Age**				
<18	41 (2.01)	426.64	27.62	ANOVA test, F-statistic = 6.17, p-value: <0.01, All means are not equal
18–45	1650 (81.08)	683.98	28.85	
45+	344 (16.90)	552.95	24.65	
**Gender**				
Male	972 (52.06)	583.97	26.35	t-statistic = 4.26, p-value: <0.01, male<female
Female	895 (47.94)	733.38	30.06	
**Area**				
Rural	739 (39.58)	589.05	25.93	t-statistic = 3.47, p-value: <0.01, rural<urban
Urban	1128 (60.42)	702.64	29.57	
**Division**				
Barishal	140 (7.50)	765.46	32.14	ANOVA test, F-statistic = 6.46, p-value: <0.01, All means are not equal
Chattogram	428 (22.92)	751.40	30.83	
Dhaka	518 (27.75)	698.96	26.82	
Khulna	233 (12.48)	462.19	22.18	
Mymensingh	124 (6.64)	751.18	30.44	
Rajshahi	92 (4.93)	692.10	32.02	
Rangpur	207 (11.09)	556.79	28.51	
Sylhet	125 (6.70)	458.31	25.00	
**Education**				
No Schooling	518 (27.75)	545.16	26.16	ANOVA test, F-statistic = 13.54, p-value: <0.01, All means are not equal
Primary	656 (35.14)	615.12	27.43	
Secondary	550 (29.46)	751.67	30.26	
Above Secondary	143 (7.66)	898.93	30.25	
**Profession**				
Service & sales	339 (18.16)	775.42	30.65	ANOVA test, F-statistic = 5.64, p-value: <0.01, All means are not equal
Craft & trade	626 (33.53)	695.70	27.89	
Plant & machine operator	89 (4.77)	532.64	25.33	
Elementary occupation	813 (43.550)	592.99	27.32	

**Fig 4:**
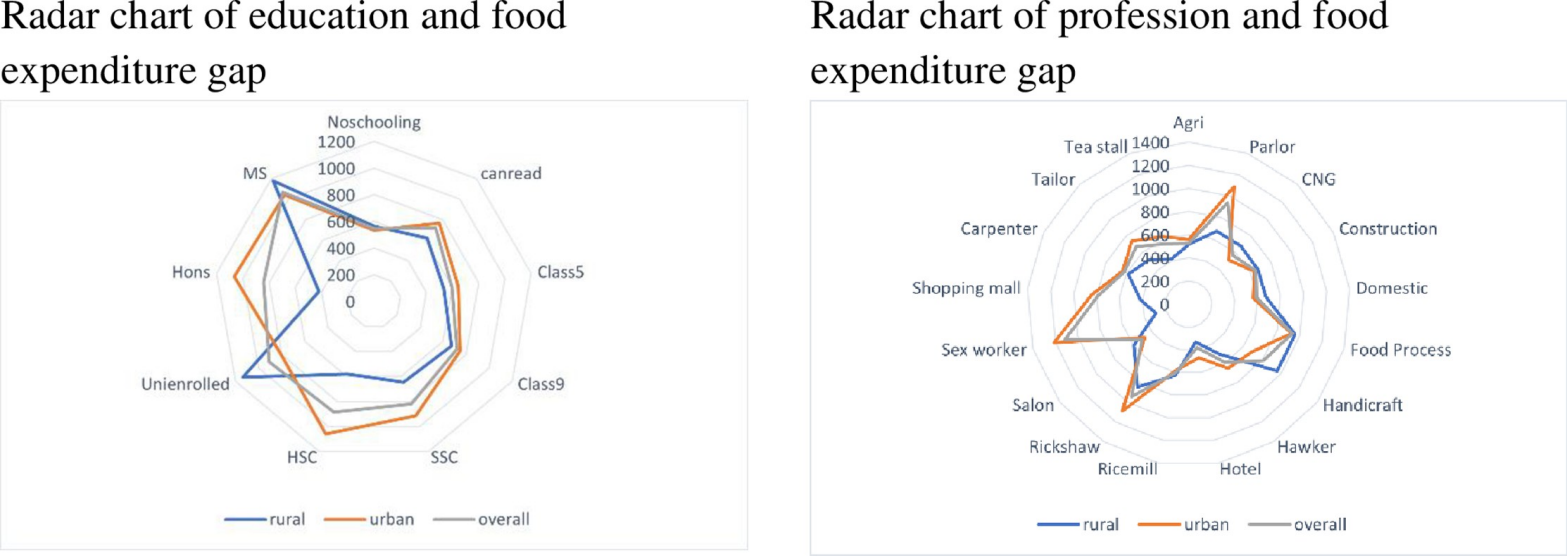
Radar charts of education and profession with the food expenditure gap.

The food expenditure gap from February to June was delineated in detail in Fig 4 for different educational levels and professions. Higher educated workers experienced a higher downfall in food expenditure. Workers involved in the agricultural sector had, however, a lesser reduction in food expenditure, whereas sex workers, rickshaw pullers, people involved in food processing and parlor had a bitter experience in food expenditure downturn.

Table 3 revealed that most of the informal workers (98%) faced an income drop in June compared to February [6, 9]. Rural females and urban males showed a bit higher rate of income decline for the lockdown.

**Table 3:** Frequency and percentages of the income gap between February and June 2020 according to area and gender.

Income gap Feb-June	Rural		Urban		Total
	Male	Female	Male	Female	
Increase	12 (3%)	2 (0.7%)	10 (2%)	19 (3%)	43 (2%)
Decrease	427 (97%)	298 (99.3%)	523 (98%)	576 (97%)	1824 (98%)
Total	439 (24%)	300 (16%)	533 (28%)	595 (32%)	1867 (100%)

Table 4 shows that 95% of the informal workers had to cut their food expenditure due to the pandemic. However, for 5% of the workers, food expenditure increased in June compared to that of February. Urban male workers had a higher drop in food expenditure compared to that of their counterparts.

**Table 4:** Frequency and percentages of the food expenditure gap between February and June 2020 according to area and gender.

Food expenditure gap Feb-June	Rural		Urban		Total
	Male	Female	Male	Female	
Increase	26 (6%)	18 (6%)	20 (4%)	31 (5%)	95 (5%)
Decrease	413 (94%)	282 (94%)	513 (96%)	564 (95%)	1772 (95%)
Total	439 (24%)	300 (16%)	533 (28%)	595 (32%)	1867 (100%)

Table 5 presented the paired t-tests checking the statistical significance between pre and post-COVID income and food expenditure downfalls. The differences in income and food expenditure from February to June were significant at a 1 percent level of significance. The average weekly food expenditure was BDT 2,173 (US$ 26) in February, which decreased to BDT 1,515 (US$ 18) in June. Average food expenditure decreased at a higher percentage (31%) for urban workers compared to that (29%) for rural workers. The income elasticity of food expenditure downfall was measured as 0.44. As a coping strategy during a crisis moment, it was hypothesized that vulnerable people would reduce their food consumption expenditure. For the informal sector during COVID-19, the measured elasticity was less than 1, which means inelastic. It seems logical for necessary goods such as staple food to be inelastic. BBS [57] estimated that 47.69% of the monthly household consumption expenditure was distributed only for food and beverage in Bangladesh. This study used per-capita worker’s food expenditure; overall household consumption expenditure could not, however, be collected during the survey.

**Table 5:** Paired t-test for income and food expenditure gaps between February and June 2020.

Variable	Overall mean (standard error)	Test statistic	Rural mean (standard error)	Test statistic	Urban mean (standard error)	Test statistic
Income in February	10613.92 (185.64)	t-statistic = 44.86*** February > June	9977.47 (276.77)	t-statistic = 28.53*** February > June	11030.88 (247.35)	t-statistic = 34.92*** February > June
Income in June	3785 (110.14)		3716.68 (173.53)		3829.77 (142.55)	
Food expenditure in February	2172.91 (32.41)	t-statistic = 36.72*** February > June	2024.91 (50.48)	t-statistic = 19.17*** February > June	2270.04 (42.01)	t-statistic = 32.44*** February > June
Food expenditure in June	1515.23 (25.99)		1435.60 (42.15)		1567.40 (32.91)	

*** means test-statistic is significant at 1% level of significance.

This study comprised five different models in Table 6 to find the best-fitted model with a list of covariates for income downfall. S1 Appendix included the forward selection models for covariate selection and revealed that Model 1 in Table 6 possessed the highest adjusted-*R*^2^ with the lowest AIC and BIC. Regression models showed low adjusted-*R*^2^. For a cross-sectional data, adjusted-*R*^2^ is not an absolute indicator of goodness of fit [58, 59]. Rather, it is a relative measure, may be a non-linearity exists in the model. The estimated residual graphs in this study depicted the normal distribution after fitting each of the models. The category with the lowest percentage of income downfall (except education) was considered as the base or reference category. Justifications of the models were explained in the methodology section. For income downfall, the OLS Model 1 and the zero-inflated probit models showed a better fit with the lower AIC and BIC, and also coefficients are found significant. As the age increased by one year, on average, workers had faced BDT 321 (US$ 4) downfall with a 1% level of significance. But after a certain age, workers experienced a smaller income downfall. Female workers, on average, had a lower income downturn than male workers, as they were paid less, but the probability of income shortfall was about two times higher for the pandemic. Due to the lockdown, workers from Barishal and Rangpur divisions had shown consistently higher income downfalls than that of the Khulna division. More educated workers displayed more income gaps in amount—on an average more than a BDT 2,284 (US$ 27) income gap for the workers with above secondary level education compared to that of the no-schooling one. At the same time, higher educated workers had about a two-times lower chance of income downfall compared to that of the no-schooling workers. Compared to the plant and machine operators, workers involved with service and sales had on an average BDT 2,248 (US$ 26) higher income gap from February to June. As the agriculture sector was not completely shut down during the pandemic [10], only a few job sectors were affected significantly.

**Table 6:** Multivariate regression analysis and determinants of income downfall.

Variables	Model 1 (OLS, Dep. var. income gap: Feb.–June)	Model 2a (Heckman 1st stage model: Dep. var. income gap: Feb.–June)	Model 2b (Heckman selection model: Dep. var. income gap: Feb.–June)	Model 3 (OLS, Dep. var.: percent change in income, Feb. to June)	Model 4 (Probit model, Dep. var.: 1 for income downfall, 0 for income increase from Feb. to June)	Model 5 (Zero-inflated Probit model, Dep. var.: 1 for income downfall, 0 for income increase from Feb. to June)
Age	320.64***	320.51***	0.002	-0.409	0.061*	0.095**
	(87.01)	(86.58)	(0.023)	(0.487)	(0.035)	(0.044)
Age-square	-3.68***	-3.68***	-.00007	0.004	-0.0007*	-0.0014**
	(1.09)	(1.09)	(0.0003)	(0.006)	(0.0004)	(0.0005)
Gender						
Female	-1104.69***	-1106.27***	-0.33***	9.02***	0.028	1.835***
	(310.27)	(308.81)	(0.092)	(1.73)	(0.149)	(0.323)
Area						
Urban	794.33**	793.87**	0.08	1.11	0.165	0.113
	(321.46)	(319.92)	(0.091)	(1.80)	(0.149)	(0.190)
Division						
Barishal	1691.86**	1692.84**	-0.353	8.01**	0.045	1.19***
	(674.07)	(670.81)	(0.221)	(3.77)	(0.278)	(0.389)
Chattogram	1930.69***	1929.34***	-0.306	4.71	-0.044	-0.158
	(516.90)	(514.42)	(0.182)	(2.89)	(0.205)	(0.241)
Dhaka	2142.16***	2142.17***	-0.344	4.76*	0.195	0.253
	(506.72)	(504.27)	(0.179)	(2.83)	(0.212)	(259)
Mymensingh	2987.69***	2987.68***	-0.089	6.25	0.066	0.110
	(703.31)	(699.90)	(0.273)	(3.93)	(0.299)	(0.356)
Rajshahi	1141.36	1142.16	-0.525	10.61**	0.467	0.323
	(774.24)	(770.50)	(0.229)	(4.33)	(0.424)	(0.507)
Rangpur	1309.68**	1310**	-0.587***	11.91***	0.727*	0.803*
	(608.21)	(605.26)	(0.189)	(3.40)	(0.389)	(0.456)
Sylhet	2885.51***	2885.34***	-0.886***	12.58***	0.284	0.694
	(698.13)	(694.76)	(0.197)	(3.90)	(0.332)	(0.443)
Education						
Primary	632.47*	632.17*	0.326***	-2.33	-0.039	-0.178
	(376.71)	(374.89)	(0.106)	(2.10)	(0.175)	(0.219)
Secondary	1495.56***	1495.40***	0.217*	-4.27	-0.199	-0.556**
	(408.72)	(406.74)	(0.112)	(2.28)	(0.178)	(0.222)
Above Secondary	2284.11***	2283.89***	0.567***	-3.36	-0.109	-1.64***
	(627.08)	(624.05)	(0.211)	(3.51)	(0.318)	(0.499)
Profession						
Service & sales	2247.53***	2247.76***	0.157	3.28	0.172	0.282
	(761.50)	(757.82)	(0.187)	(4.26)	(0.350)	(0.418)
Craft & trade	-1421.94*	-1422.72**	0.638***	-2.04	-0.089	-1.24***
	(728.89)	(725.36)	(0.185)	(4.08)	(0.332)	(0.440)
Elementary occupation	-1020.13	-1020.12	0.270	-0.249	-0.057	-0.640*
	(706.51)	(703.09)	(0.169)	(3.95)	(0.319)	(0.378)
Selection (BRAC beneficiary = 1)			0.082			
			(0.096)			
Adjusted-*R*^2^	0.091	--	--	0.021	--	--
rho	--	--	-0.049	--	--	--
			(0.145)			
Pseudo-*R*^2^	--	--	--	--	0.037	--
Wald statistic	--	--	--	--	--	47.29
AIC	37964.77	--	39131.85	18604.23	429.99	427.82
BIC	38064.35	--	39255.45	18703.81	529.57	526.69

Reference category: Gender: Male, Area: Rural, Division: Khulna, Education: No schooling, Profession: Plant & machine operator; * for 10%,

** for 5%,

*** for 1% level significance respectively; parenthesis indicates the standard error.

Table 7 explained five different models for food expenditure downturn and according to AIC, BIC and significance of coefficients, Model 1 with OLS and Model 3 with percent changes showed a better fit. Heckman specification model delineated a similar pattern to the OLS model and the estimated rho was insignificant. The covariate age depicted an increasing pattern of food expenditure shortfall, but due to the nonlinear impact of age, the food expenditure gap reduced after a while. Female workers had a greater expenditure gap (BDT 135, US$ 2) in amount and percent change (3.33) compared to that of their male counterparts. Urban informal workers faced a larger food expenditure downfall than that of the rural workers both in amount and in percentage (4.06%) with a high statistical significance. Workers from the Khulna division had significantly lower food expenditure declines than those of the other divisions. On an average, workers with above secondary level education had a higher food expenditure gap of BDT 345 (US$ 4) compared to that of the no-schooling workers at 1% level of significance. Association between profession and food expenditure gap was statistically insignificant. Overall the covariates outlined a similar kind of association and significance levels for both income and food expenditure downfall.

**Table 7:** Multivariate regression analysis and determinants of food expenditure downfall.

Variables	Model 1 (OLS, Dep. var. food exp. gap: Feb.–June)	Model 2a (Heckman 1st stage model: Dep. var. food exp. gap: Feb.–June)	Model 2b (Heckman selection model: Dep. var. food exp. gap: Feb.–June)	Model 3 (OLS, Dep. var.: percent change in food exp., Feb. to June)	Model 4 (Probit model, Dep. var.: 1 for food exp. downfall, 0 for food exp. increase from Feb. to June)	Model 5 (Zero-inflated Probit model, Dep. var.: 1 for food exp. downfall, 0 for food exp. increase from Feb. to June)
Age	27.92***	27.17***	0.076	0.880*	0.008	0.008
	(10.48)	(9.72)	(0.051)	(0.496)	(0.028)	(0.030)
Age-square	-0.334**	-0.322***	-0.0009	-0.011*	-0.0001	-0.0002
	(0.132)	(0.122)	(0.0006)	(0.006)	(0.0003)	(0.0003)
Gender						
Female	134.89***	131.57***	-0.910***	3.33*	0.162	0.172
	(37.37)	(34.69)	(0.298)	(1.77)	(0.105)	(0.137)
Area						
Urban	71.68*	77.02**	0.041	4.06**	0.176	0.191
	(38.72)	(36.16)	(0.237)	(1.83)	(0.107)	(0.158)
Division						
Barishal	312.73***	302.35***	-4.91***	10.17***	0.256	0.276
	(81.20)	(76.63)	(1.16)	(3.84)	(0.242)	(0.293)
Chattogram	286.83***	279.18***	-4.66***	7.99***	0.115	0.126
	(62.26)	(59.10)	(1.08)	(2.95)	(0.173)	(0.202)
Dhaka	217.29***	206.69***	-4.51***	3.23	0.013	0.017
	(61.04)	(57.92)	(1.13)	(2.89)	(0.167)	(0.182)
Mymensingh	297.12***	293.95***	-4.81***	8.95**	-0.061	-0.064
	(84.72)	(80.82)	(1.43)	(4.01)	(0.217)	(0.238)
Rajshahi	226.88**	245.36***	-4.95***	9.68**	0.102	0.115
	(93.26)	(86.85)	(1.11)	(4.42)	(0.259)	(0.295)
Rangpur	138.34*	132.84*	-5.07***	7.24**	0.162	0.173
	(73.26)	(68.24)	(1.08)	(3.47)	(0.204)	(0.234)
Sylhet	-11.64	-29.12	-4.79***	2.38	0.219	0.238
	(84.09)	(76.39)	(1.13)	(3.98)	(0.245)	(0.295)
Education						
Primary	64.26	75.09*	0.232	1.22	0.126	0.136
	(45.38)	(42.05)	(0.367)	(2.15)	(0.133)	(0.163)
Secondary	186.87***	195.33***	-0.352	3.43	0.068	0.071
	(49.23)	(45.49)	(0.321)	(2.33)	(0.136)	(0.148)
Above Secondary	345.01***	355.74***	-0.163	3.27	0.106	0.106
	(75.54)	(70.94)	(0.492)	(3.58)	(0.199)	(0.215)
Profession						
Service & sales	141.15	119.98	0.821**	3.32	0.095	0.104
	(91.73)	(84.31)	(0.378)	(4.34)	(0.226)	(0.254)
Craft & trade	69.57	53.89	1.39***	0.458	0.463**	0.496
	(87.80)	(80.82)	(0.409)	(4.16)	(0.224)	(0.350)
Elementary occupation	50.64	42.80	1.02***	1.35	0.308	0.332
	(85.11)	(77.98)	(0.336)	(4.03)	(0.212)	(0.295)
Selection (BRAC beneficiary = 1)			0.142			
			(0.228)			
Adjusted-*R*^2^	0.047	--	--	0.008	--	--
rho	--	--	-0.063	--	--	--
			(0.290)			
Pseudo-*R*^2^	--	--	--	--	0.027	--
Wald statistic	--	--	--	--	--	4.08
AIC	30062.09	--	32602.03	18676.38	766.39	768.23
BIC	30161.67	--	32725.64	18775.95	865.97	867.92

Reference category: Gender: Male, Area: Rural, Division: Khulna, Education: No schooling, Profession: Plant & machine operator; * for 10%,

** for 5%,

*** for 1% level significance respectively; parenthesis indicates the standard error.

**Fig 5:**
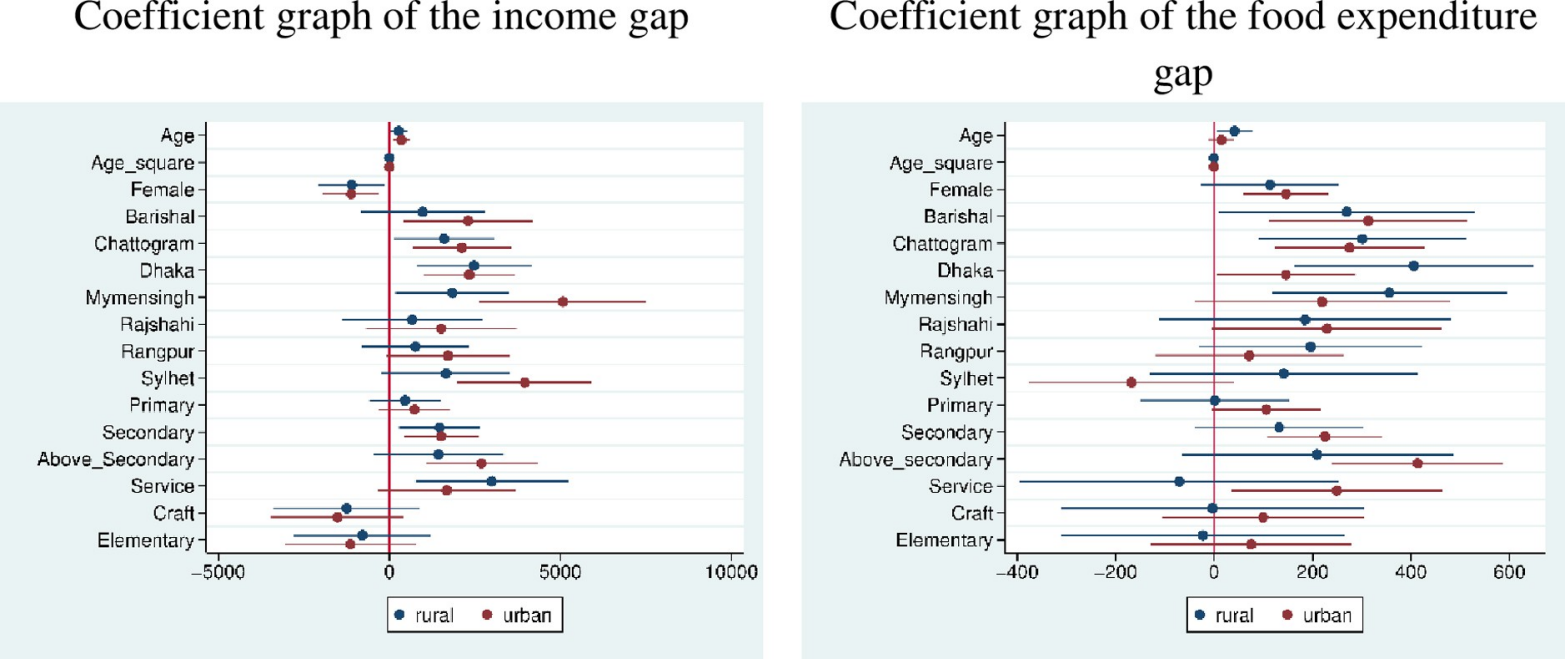
Coefficient graphs of the income and food expenditure gaps.

Fig 5 exhibited the coefficient graphs of Model 1 differentiating the rural-urban confidence intervals. In the equation of income gap, informal workers in Mymensingh and Sylhet showed a high interval for the urban areas. In comparison, food expenditure dwindled in Dhaka and Mymensingh with a large confidence interval for the rural areas. As Model 1 in Tables 6 and 7 did not include the confidence intervals, only depicted the coefficients, standard errors, and significance, Fig 4 thus explained the interval changes explicitly.

A validation test (Pearson *χ*^2^) was performed in Table 8 to check the validity of the informal workers’ perception and change in income, which depicts that 97.7% experienced income downfall. In comparison, around 85% reportedly faced problems in service, business, and production during COVID-19. The chi-square test revealed a significant association (at 95% confidence interval, p-value 0.019) between the workers experiencing problems (in running business, service, and production process) and income downfall. About half of the workers (46%) reported unemployment. Half of the respondents (58% male and 42% female) mentioned unavailability of work due to movement restrictions. Among the employed, almost all urban workers reported getting less payment. Above 90% of rural workers also reported getting less payment during the lockdown period. More females than males mentioned being unemployed during the lockdown (50% and 42%, respectively). About 27% of the workers struggled to pay their rents for accommodation during the lockdown time. Around 50% of the respondents borrowed money as a coping strategy, while 19% coped by depleting savings [12]. Around 44% of informal workers, however, received Government or NGO aids or assistance during the pandemic. Those who received any immediate aid or assistance from NGOs were two-fold more in percentage than those receiving assistance from Government organizations. An alarming finding was, however, that about 89% of respondents suffered severe or moderate mental health issues due to financial loss. Female (92%) and urban (91%) workers suffered from mental stress more in percentages than their counterparts.

**Table 8:** Income downfall and perception validity test.

Income gap (Feb-June)	Perception (faced problems)		Total
	Yes	No	
Decrease	1583 (84.79%)	241 (12.91%)	1824 (97.70%)
Not decrease	32 (1.71%)	11 (0.69%)	43 (2.30%)
Total	1615 (86.50%)	252 (13.50%)	1867 (100%)

**Fig 6:**
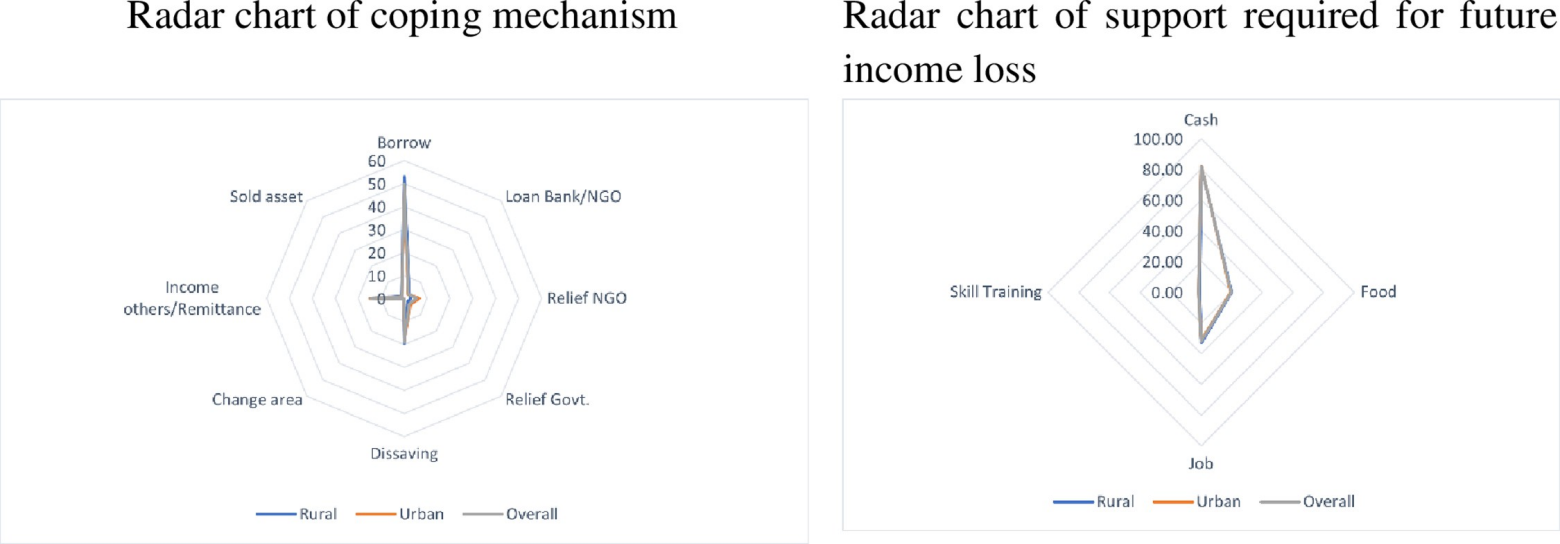
Coping mechanism for current shock and support required for future shock of informal workers.

Fig 6 showed that around half (49.8%) of the respondents borrowed money from neighbors and relatives as a coping strategy. During COVID-19 pandemic social network played the major role. Among the borrowers rural percentage is a bit higher than that of the urban informal workers. About 3% took loans from banks/NGOs, and here rural informal workers were higher in percentage. The second major coping strategy was dissaving, about 19% informal workers coped by depleting savings [12] and the percentage is higher in rural areas. Urban informal workers received more Government and NGOs’ assistance (10.2%) compared to that of their rural counterparts (5%). Around 15% informal workers coped with the earning of other family members. Remittance was the third major coping strategy. When asked how to cope with the future income loss, about 81% of the informal workers, especially the urban ones, mentioned getting cash assistance. Around 31% responded on job support and 19% for food items. The preference sequence being the same, the percentages were, however, higher for rural informal workers. Only a few (1.6%) respondents opted for skill-training.

## 4. Discussion and conclusion

The economic and social shock induced by the COVID-19 pandemic is a universal phenomenon that has continued to affect the lives of all sectors of society. The impacts, however, varied with the urban-rural nature, gender, locations, level of education, types of work, and age of the informal sector workers in Bangladesh. The absence of official protection and recognition, social security system, and restricted benefits from institutional sources has pushed informal sector workers to a precarious condition [8,12, 60]. Another finding of this study was that the pandemic had less impact on the income of the rural informal workers, especially those who were involved in agriculture-based employment. On the contrary, urban informal sector workers were the worst hit by the pandemic as they faced the restriction of movements in towns during the lockdown. As a result, average per capita household consumption was lower in urban areas compared to that of the rural areas, and the increase in the poverty rate was higher in urban areas [29]. Urban informal sector workers mainly live in poor conditions with their mere amount of daily income, and they were worst hit by the pandemic [61].

The COVID-19 impact on the informal sector workers has a gender face. Analyses showed that the crisis had hit the rural women, a dominant figure in the informal economy, hardest in terms of unemployment and loss of income. However, the male workers in urban areas were more affected than their female counterparts [17, 56]. The income generated by rural females has dropped a bit more compared to that of the urban females. The agri-processing factories stopped operating their business during the lockdown, particularly with the value chains where women were involved and thus lost their income opportunities [56]. This study also found that higher education might not guarantee additional financial security during a pandemic if someone is involved in the informal sector, even though the sample size was small. While the strict lockdown and social distancing have led to the decrease in demand for beauty parlor, hotel and restaurant services, interestingly, the study has found out that workers in the sanitation and home service professions have been least affected. The COVID-19 situation necessitated the continuation of caregiving and cleaning services, provided primarily by women workers [62]. It highlights that practical competencies in technical and vocational skills regardless of crisis are crucial, and vocational skill-based education can be a safety net for the economy. Thus, a new focus on vocational education may contribute to a growing informal economy.

The income drop of the informal sector workers impacted their well-being. Study findings suggested that they had to decrease more than one-fourth proportion of their food expenditure between February and June 2020. Urban informal workers had to reduce their food expenditure than that of their rural counterparts both in amount and in percentage. There are no alternatives but to enhance the capacity building of the informal workers and set a proper labor right to fight against such a crisis [63]. Interestingly, these people are not targeted in traditional social safety nets and in other initiatives funded by the public exchequer. The findings may help policymakers reexamine the existing programs and take initiatives and tweak such programs so that the most affected are targeted and offered the right kind of support. An appropriate database, Government-NGO-based combined social safety net and mobilizing the micro-finance institute may facilitate the informal sector workers to cope with future distress [64]. One quick lead from this study is to boost employment-oriented policies rather than focus merely on the growth-oriented policies to adopt in the post-COVID era [65]. Even though transitioning to a new normal is being confronted with numerous challenges by the informal workers such as socio-economic conditions and socio-emotional state, re-evaluation for preparedness of the current structure in the informal sector for future scenarios and challenges is imperative.

This study was based on telephone interviews, conducted during the lockdown. Therefore, a short questionnaire was used and thus, the study failed to extract all the information of the respondents. There was no question to differentiate the wage earners and self-employed. A second-round panel study should be commissioned to get the answers to the unresolved questions.

## Supporting information

S1 Data (ZIP)

S1 Appendix (DOCX)

## References

[1] WHO (2022). https://covid19.who.int/ Retrieved on 26 February, 2022.

[2] Harvey J. (2022). Covid-19’s Toll on the World’s Informal Workers. New Labor Forum. 31(1):60–68. doi: 10.1177/10957960211062873

[3] World Bank (2020a). Bangladesh Must Ramp Up COVID-19 Action to Protect its People, Revive Economy, Retrieved on 9 June, 2021. https://www.worldbank.org/en/news/press-release/2020/04/12/bangladesh-must-act-now-to-lessen-covid-19-health-impacts

[4] ILO (2020a). International Labour Organization. As Job Losses Escalate, Nearly Half of Global Workforce at Risk of Losing Livelihoods. Press release, Retrieved on 11 June, 2021. https://www.ilo.org/global/about-the-ilo/newsroom/news/WCMS_743036/lang—en/index.htm

[5] Onyekwena, C., & Ekeruche, M. A. (2020). Understanding the impact of the COVID-19 outbreak on the Nigerian economy, Retrieved on 14 June, 2021. https://Www.Brookings.Edu/Blog/Africa-in-Focus/2020/04/08/Understanding-the-Impact-of-the-Covid-19-Outbreak-on-the-Nigerian-Economy/ (Accessed 21.04.2021).

[6] Narula R. (2020). Policy opportunities and challenges from the Covid-19 pandemic for economies with large informal sectors. Journal of International Business Policy, 3, 302–310. 10.1057/s42214-020-00059-5

[7] Guo F., Huang Y., Wang J., & Wang X. (2022). The informal economy at times of COVID-19 pandemic. China Economic Review, 101722. 10.1016/j.chieco.2021.101722 35058681 PMC8609671

[8] Pitoyo, A., Aditya, B., & Amri, I. (2020). The impacts of COVID-19 pandemic to informal economic sector in Indonesia: Theoretical and empirical comparison, Retrieved on 15 June, 2021. E3S Web of Conferences 200, 03014. 10.1051/e3sconf/202020003014

[9] Kansiime M. K., Tambo J. A., Mugambi I., Bundi M., Kara A., & Owuor C. (2020). COVID-19 implications on household income and food security in Kenya and Uganda: Findings from a rapid assessment. World Development, 105199. 10.1016/j.worlddev.2020.105199 32982018 PMC7500897

[10] Mahmud M., & Riley E. (2020). Household response to an extreme shock: Evidence on the immediate impact of the COVID-19 lockdown on economic outcomes and well-being in rural Uganda. World Development, 140, 105318. 10.1016/j.worlddev.2020.105318 34548741 PMC8446716

[11] Stoop N., Desbureaux S., Kaota A., Lunanga E., & Verpoorten M. (2020). Covid-19 vs. Ebola: Impact on households and small businesses in North Kivu, Democratic Republic of Congo. World Development, 105352. 10.1016/j.worlddev.2020.105352 34548742 PMC8446712

[12] Komin W., Thepparp R., Subsing B., & Engstrom D. (2020). Covid-19 and its impact on informal sector workers: A case study of Thailand. Asia Pacific Journal of Social Work and Development, 31(1), 1–9. 10.1080/02185385.2020.1832564

[13] Dang H.-A., & Nguyen C. V. (2020). Gender inequality during the COVID-19 pandemic: Income, expenditure, savings, and job loss. World Development, 140(2). 10.1016/j.worlddev.2020.105296 PMC8446715 34548740

[14] Romanello, M. (2022). Covid-19 and the Informal Sector. In: Papyrakis E. (eds) COVID-19 and International Development. Springer, Cham. 10.1007/978-3-030-82339-9_7

[15] Leyva, G., & Urrutia, C. (2020). Informal Labor Markets in Times of Pandemic: Evidence for Latin America and Policy Options. Unpublished Manuscript, ITAM.

[16] BRAC (2020). Bangladesh Rural Advancement Committee. Rapid food and income security assessment: How are BRAC International volunteers and programme participants coping with COVID-19. Retrieved on 15 June, 2021, https://covid19.bracinternational.nl/wp-content/uploads/2020/04/Covid-FS-Rapid-Assessment-BI_20200404.pdf

[17] ILO (2020b). COVID-19 Crisis and the Informal Economy. ILO brief, Retrieved on June 12, 2021. https://www.ilo.org/wcmsp5/groups/public/@ed_protect/@protrav/@travail/documents/briefingnote/wcms_743623.pdf

[18] Zollmann J., Ng’weno A., Gachoka A., & Wanjal C. (2020). When Hustling Fails: The Impact of Coronavirus Mitigation Efforts on Ordinary People’s Livelihoods. BFA Global Financial Diaries, Retrieved on 9 June, 2021. https://bfaglobal.com/financial-diaries/insights/when-hustling-fails-the-impact-of-coronavirus-mitigation-efforts-on-ordinary-peoples-livelihoods/

[19] Wallace, M. (2020). A Snapshot of Indebtedness During the COVID-19 Crisis in Myanmar. ONOW Myanmar, Medium, Retrieved on 10 June, 2021. https://medium.com/opportunities-now-myanmar/a-snapshot-of-indebtedness-during-the-covid-19-crisis-in-myanmar-4b9911b6b14e

[20] UN Human Rights (2020). COVID-19 and the Human Rights of LGBTI People. Office of the High Commissioner for Human Rights, Topics in Focus, Retrieved on 11 June, 2021. https://www.ohchr.org/Documents/Issues/LGBT/LGBTIpeople.pdf

[21] OAS (2020). Organization of American States. COVID-19: The suffering and Resilience of LGBT Persons Must Be Visible and Inform The Actions of States. Press release. OAS, Retrieved on 14 June, 2021. https://www.oas.org/en/iachr/media_center/PReleases/2020/110A.pdf

[22] Zambrano, A., Montoya, D., Alvarez, A., & Zuleta, H. (2021). The Role of the Informal Sector in the COVID Crisis: A Cushion or an Amplifier? Retrieved on 31 January, 2022. https://economia.uniandes.edu.co/sites/default/files/seminariocede/2021/The-Role-of-the-Informal-Sector-in-the-COVID-crisis.pdf

[23] WIEGO (2020). Women in Informal Employment. Globalizing and Organizing. Impact of Public Health Measures on Informal Workers’ Livelihoods: Rapid Assessment, Retrieved on 10 June, 2021. Manchester, UK: WIEBO, April. https://www.wiego.org/resources/impact-public-health-measures-informal-workers-livelihoods-rapid-assessment

[24] BBS (2020a). Statistical Yearbook Bangladesh 2019. Statistics & Informatics Division (SID), Ministry of Planning, Government of the People’s Republic of Bangladesh. Retrieved on 17 June, 2021, http://www.bbs.gov.bd/site/page/29855dc1-f2b4-4dc0-9073-f692361112da/Statistical-Yearbook

[25] ADB (2012). Asian Development Bank. The informal sector and informal employment in Bangladesh. Retrieved on 16 June, 2021, https://www.adb.org/sites/default/files/publication/30084/informal-sector-informal-employment-bangladesh.pdf

[26] Khondker B. H. (2019). Insights into the informal sector of Bangladesh. The Financial Express. Retrieved on 14 June, 2021. https://www.thefinancialexpress.com.bd/views/insights-into-the-informal-sector-of-bangladesh

[27] BBS (2017). Labour Force Survey 2016–2017. Ministry of Planning, Retrieved on 17 June, 2021, GoB. http://data.bbs.gov.bd/index.php/catalog/200

[28] Kumar B., & Pinky S. D. (2020). Addressing economic and health challenges of COVID-19 in Bangladesh: Preparation and response. Journal of Public Affairs. 10.1002/pa.2556 33349743 PMC7744919

[29] Genoni, M. E., Khan, A. I., Nandini, K., Nethra, P., & Wameq, R. (2020). Losing livelihoods: The labour market impacts of COVID-19 in Bangladesh. The World Bank. Washington, DC. Retrieved on June 17, 2021, https://openknowledge.worldbank.org/bitstream/handle/10986/34449/Losing-Livelihoods-The-Labor-Market-Impacts-of-COVID-19-in-Bangladesh.pdf

[30] Islam S., Islam R., Mannan F., Rahman S., & Islam T. (2020). COVID-19 pandemic: An analysis of the healthcare, social and economic challenges in Bangladesh. Progress in Disaster Science, 8(2020). 10.1016/j.pdisas.2020.100135 34173450 PMC7669476

[31] World Bank (2020b). Losing livelihoods: The labour market impacts of COVID- 19 in Bangladesh. World Bank, Retrieved on 9 June, 2021. http://documents1.worldbank.org/curated/en/475551600152674960/ppdf/Losing-Livelihoods-The-Labor-Market-Impacts-of-COVID-19-in-Bangladesh.pdf

[32] Rahman, H. Z., & Matin, I. (2020). Livelihoods, coping, and support during COVID-19 crisis. Dhaka, BRAC Institute of Governance and Development, Retrieved on 12 June, 2021. https://bigd.bracu.ac.bd/wp-content/uploads/2020/06/PPRC-BIGD-Final-April-Survey-Report.pdf

[33] Barkat, A. (2020). Coronavirus-19: Shombhaboo Onishchoyota O Koronio Kalpo Chitro. Retrieved on September 10, 2021, https://www.hdrcbd.com/wpcontent/uploads/2020/04/CoronaVirus%2019%20Somvavbo%20Onishchoyota%20O%20Koronio%20KolpoChitro-09-04-20%20L%20(1).pdf

[34] Islam R. (2020). The Impact of COVID-19 on Employment in Bangladesh: Pathway to an Inclusive and Sustainable Recovery. Dhaka: Bangladesh Institute of Labour Studies.

[35] Imran, S., Huda, A., Mahmud, P., Reza, M., & Imran, M. (2020). Impact of COVID-19 on Low-income Professions. Innovision, Retrieved on 11 June, 2021. http://innovision-bd.com/covid-19/COVID-19_Digest-12_Low-Income-Groups.pdf

[36] Shammi M., Bodrud-Doza M., Islam A. R. M. T., & Rahman M. M. (2021). Strategic assessment of COVID-19 pandemic in Bangladesh: Comparative lockdown scenario analysis, public perception, and management for sustainability. Environment, Development and Sustainability, 23, 6148–6191. 10.1007/s10668-020-00867-y 32837281 PMC7368637

[37] CPD-BILS (2021). Impact of COVID-19 on the Labor Market. Retrieved on 10 September 2021. https://cpd.org.bd/wp-content/uploads/2021/04/Presentation-on-Impact-of-COVID-19-on-Labour-Market.pdf

[38] Wasima S. & Rahman M.N. (2022). Economic Vulnerability of the Underprivileged during the COVID Pandemic: The Case of Bangladeshi Domestic Workers. Journal of Social Service Research. 10.1080/01488376.2022.2029799

[39] Ali, M., Bhuiyan, M. (2020). Around 1. 7m youths may lose jobs in 2020 for pandemic. The Business Standard. Retrieved on 12 June, 2021, https://tbsnews.net/bangladesh/around-17m-youths-may-lose-jobs-2020-pandemic-121351

[40] Islam, R., & Jahangir, A. R. (2020). Corona fallout spells disaster for millions of poor Bangladeshis: Economists, Retrieved on 15 June, 2021. United News of Bangladesh. https://unb.com.bd/category/special/corona-fallout-spells-disaster-for-millions-of-poor-bangla-deshis-economists/48533

[41] Kamruzzaman, M. (2020). Coronavirus: Poor income drops 80% in Bangladesh. Anadolu Agency. Retrieved on 13 June, 2021. https://www.aa.com.tr/en/asia-pacific/coronavirus-poor-income-drops-80-in-bangladesh/1808837

[42] Raihan, S., & Bidisha, S. H. (2018). Female employment stagnation in Bangladesh. The Asia Foundation, Retrieved on 12 June, 2021. https://asiafoundation.org/wp-content/uploads/2018/12/EDIG-Female-employment-stagnation-in-Bangladesh_report.pdf

[43] Bhattacharjee, J. (2020). Bangladesh: COVID-19 badly impacts garment industry. Observer Research Foundation (ORF). Retrieved on June 16, 2021, https://www.orfonline.org/research/bangla-desh-covid19-badly-impacts-garments-industry-65275/

[44] Mahmud M. A. F., Rafi A. H., Noman M. N., Shaekh A. A., & Rakibuzzaman M. (2020). COVID-19 impact on Bangladesh economy. Lanka Bangla Asset Management, Retrieved on 12 June, 2021. https://www.arx.cfa/-/media/regional/arx/post-pdf/2020/06/22/covid-19-impact-on-bangladesh-economy.ashx

[45] Shawon A. A. (2020). Tk760cr Virus Aid Package Rolled Out for Daily Wage Earners. Dhaka Tribune, Retrieved on June 14, 2021. https://www.dhakatribune.com/health/coronavirus/2020/04/13/unemployment-due-to-covid-19-govt-sanctions-tk760cr-for-daily-wage-earners

[46] Das T. K. (2020). Theft of Rice Meant for Poor Raises Concern. Business Standard, Retrieved on 15 June, 2021, https://www.newagebd.net/article/103891/theft-of-rice-meant-for-poor-raises-concern

[47] Demirguc-Kunt A., Klapper L., Singer D., Ansar S., & Hess J. (2018). Global Findex Database 2017: Measuring Financial Inclusion and the Fintech Revolution. Washington, D.C.: World Bank. https://openknowledge.worldbank.org/handle/10986/29510

[48] Gentilini U., Almenfi M., Dale P., Blomquist J., Natarajan H., Galicia G., et al. (2020). Social Protection and Jobs Responses to COVID- 19: A Real-Time Review of Country Measures. Living paper version 10, Retrieved on 15 June, 2021. https://www.ugogentilini.net/wp-content/uploads/2020/05/Country-SP-COVID-responses_May22.pdf

[49] Enano J. (2020). Less Than Half of Subsidy Doled Out. Philippine Daily Inquirer, Retrieved on 14 June, 2021, https://newsinfo.inquirer.net/1265668/less-than-half-of-subsidy-doled-out#ixzz6QIn9Gi24

[50] Rahman, M., Moazzem, K.G. & Hossain, S.S. (2009). Impact of the Global Economic Crisis on the Employment and Labour Market of Bangladesh A Preliminary Assessment, Labor Economics Working Papers 22303, East Asian Bureau of Economic Research. https://ideas.repec.org/p/eab/laborw/22303.html

[51] Cochran W. G. (1977). Sampling Techniques, 3rd Edition. Wiley.

[52] BBS (2020b). Consumer Price Index (CPI), Inflation Rate and Wage Rate Index (WRI) in Bangladesh. Bangladesh Bureau of Statistics (BBS), Retrieved on 7 August, 2021, https://bbs.portal.gov.bd/sites/default/files/files/bbs.portal.gov.bd/page/9ead9eb1_91ac_4998_a1a3_a5caf4ddc4c6/2020-03-03-17-01-83f67fbc363665c4c5e63c5cdf2c4f3e.pdf

[53] Greene W. H. (2017). Econometric Analysis, 8th Edition. Pearson.

[54] Gujarati D. N., Porter D. C., & Gunasekar S. (2017). Basic econometrics, 5th Edition. McGraw-Hill Education.

[55] BSCO (2020). Bangladesh Standard Classification of Occupations. Volume II. Retrieved on 25 August, 2021, http://bbs.portal.gov.bd/sites/default/files/files/bbs.portal.gov.bd/page/745673c8_c7ed_49bc_a4e2_e7b05fe7a9d4/2021-06-09-09-31-39713fd0bcba7a89017121c485716b2b.pdf

[56] FAO (2020). Gendered impacts of COVID-19 and equitable policy responses in agriculture, food security and nutrition. Rome, Italy: Food and Agriculture Organization of the United States. Retrieved on from https://reliefweb.int/sites/reliefweb.int/files/resources/CA9198EN.pdf

[57] BBS (2016). Bangladesh Bureau of Statistics. Household Income and Expenditure Survey 2016 Report. Retrieved on 27 August, 2021, https://bbs.portal.gov.bd/sites/default/files/files/bbs.portal.gov.bd/page/a1d32f13_8553_44f1_92e6_8ff80a4ff82e/2020-05-15-09-25-dccb5193f34eb8e9ed1780511e55c2cf.pdf

[58] Achen C.H. (1982) Interpreting and using regression, Newbury Park: Sage University Papers.

[59] Anderson-Sprecher R. (1994). Model comparisons and R^2^. The American Statistician 48(2), 113–117.

[60] Haque M.R., Khan M.M.A., Rahman M.M., Rahman M.S., & Begum S.A. (2022). Mental health status of informal waste workers during the COVID-19 pandemic in Bangladesh. PLoS ONE 17(1): e0262141. 10.1371/journal.pone.0262141 34995288 PMC8741044

[61] Sakamoto M., Begum S., & Ahmed T. (2020). Vulnerabilities to COVID-19 in Bangladesh and a reconsideration of sustainable development goals. Sustainability, 12(13). 10.3390/su12135296 32944297

[62] UN Women (2020). COVID-19 and its economic toll on women: The story behind the numbers, Retrieved on 10 June, 2021. https://www.unwomen.org/en/news/stories/2020/9/feature-covid-19-economic-impacts-on-women

[63] Mujeri, M. K. (2020). Informal economy and economic inclusion. The Daily Star, Retrieved on 15 June, 2021. https://www.thedailystar.net/supplements/29th-anniversary-supplements/digi-tisation-and-inclusivity-taking-everyone-along/news/informal-economy-and-eco-nomic-inclusion-1869601

[64] Martínez L., Young G., Trofimoff V., Valencia I., Vidal N., Espada A.D., & Robles E. (2022). The hardships of the poorest during the COVID-19 pandemic: Data about the socioeconomic conditions and governance of informal workers. Data Brief. 40:107728. doi: 10.1016/j.dib.2021.107728 Epub 2021 Dec 16. ; PMCID: PMC8699104.65. 34977304 PMC8699104

[65] Hossain M.I. (2021). COVID-19 Impacts on Employment and Livelihood of Marginal People in Bangladesh: Lessons Learned and Way Forward. South Asian Survey. 28(1):57–71. 10.1177/0971523121995072

