# Supplementary material for: Understanding the impact of COVID-19 on the informal sector workers in Bangladesh
Source: PLoS One. 2022 Mar 31;17(3):e0266014. doi: 10.1371/journal.pone.0266014 (PMC8970377; doi:10.1371/journal.pone.0266014)
Supplement: S1 Appendix — (DOCX) [file pone.0266014.s002.docx]

**S1 Appendix. Fixed effect regression analysis and forward selection of covariates.**

| **Variable** | **Model 1** | **Model 2** | **Model 3** |
| --- | --- | --- | --- |
| Age | 6.93  (14.89) | -0.047  (14.94) | 0.76  (14.90) |
| Gender |  |  |  |
| Female |  | -1192.01***  (307.25) | -1316.84***  (308.43) |
| Area |  |  |  |
| Urban |  |  | 1089.23***  (312.94) |
| Adj. R-square | 0.001 | 0.0081 | 0.015 |
| AIC | 38145.31 | 38132.29 | 38122.19 |
| BIC | 38156.37 | 38148.89 | 39144.32 |

*for 10%, ** for 5%, *** for 1% level of significance. Reference category for Gender is male, and for Area is rural.
